# Non-Obstructive Azoospermia: Influence of PRP on Proliferation, Apoptosis, and Growth Factors of Male Germ Cells

**DOI:** 10.3390/medicina61081450

**Published:** 2025-08-12

**Authors:** Grigory Demyashkin, Vladimir Shchekin, Maya Epifanova, Tatyana Borovaya, Matvey Vadyukhin, Konstantin Gotovtsev, Petr Shegay, Andrey Kaprin

**Affiliations:** 1 Department of Digital Oncomorphology, National Medical Research Centre of Radiology, 2nd Botkinsky Pass., 3, 125284 Moscow, Russia; dr.shchekin@mail.ru (V.S.); tbor27@yandex.ru (T.B.); vma20@mail.ru (M.V.); kostya.gotovtsev@gmail.com (K.G.); dr.shegai@mail.ru (P.S.); kaprin@mail.ru (A.K.); 2 Laboratory of Histology and Immunohistochemistry, Institute of Translational Medicine and Biotechnology, I.M. Sechenov First Moscow State Medical University (Sechenov University), Trubetskaya St., 8/2, 119048 Moscow, Russia; 3 Research and Educational Resource Center for Immunophenotyping, Digital Spatial Profiling and Ultrastructural Analysis Innovative Technologies, Peoples’ Friendship University of Russia (RUDN University), Miklukho-Maklaya St., 6, 117198 Moscow, Russia; 4 Department of Urology and Operative Nephrology, Peoples’ Friendship University of Russia (RUDN University), Miklukho-Maklaya St., 6, 117198 Moscow, Russia; epifanova_maya@mail.ru

**Keywords:** spermatogenesis, non-obstructive azoospermia, PRP, growth factors

## Abstract

*Background and Objectives:* Currently, infertility is one of the major problems affecting up to 12% of couples worldwide, with more than a quarter of cases being male-related. It is assumed that Leukocyte-poor platelet-rich plasma (LP-PRP) can improve the function of germ cells and serve as a regenerative substrate as a source of biologically active substances that play an important role in the process of spermatogenesis in infertile men. We aimed to evaluate the proliferation, apoptosis, and growth factors of germ cells after the administration of LP-PRP in patients with non-obstructive azoospermia. *Materials and Methods:* The study used archival material (paraffin blocks of testicular biopsies) of patients with non-obstructive azoospermia aged 21–34 years (*n* = 41; associated diagnosis: varicocele). We confirm that no interventions or biopsies were performed as part of the study itself. They were injected bilaterally into the spermatic cord and in the region of the lower pole of the testis under ultrasound control were injected with PRP once a week for 6 weeks. Biopsies were immunohistochemical reactions with antibodies to Ki-67, Bcl-2, caspase 3 and p53, IGF-1, TGF-β, and VEGF-A. *Results:* Immunohistochemical study of testicular biopsies after LP-PRP injection revealed an increase in the number of cells stained for proliferation proteins (Ki-67) and anti-apoptosis (Bcl-2), IGF-1, TGF-β, VEGF-A; decrease caspase-3- and p53-positive cells. *Conclusions:* In LP-PRP, platelet α-granule growth factors, which are key regulators of the cell cycle of germ cells, demonstrate restoration of the proliferative-apoptotic balance, confirmed by the expression levels of Ki-67, Bcl-2, caspase 3, and p53 in patients with non-obstructive azoospermia. In human testicular biopsies, the administration of LP-PRP led to an exponential release of numerous growth factors from platelet α-granules, which, based on their regenerative properties, improved the morphological and immunohistochemical picture of the germinal epithelium in non-obstructive azoospermia.

## 1. Introduction

Infertility is a pervasive problem, affecting up to 12% of married couples worldwide. Male factors are responsible for more than a quarter of cases [1]. The causes can be either individual or combined factors, including infectious, autoimmune, and idiopathic diseases, as well as malignant neoplasms, which are often treated with radiotherapy and chemotherapy, leading to prolonged azoospermia [2,3,4]. Non-obstructive azoospermia (NOA) is a particularly intricate problem, as it is characterized by the absence of spermatozoa in the ejaculate due to spermatogenesis disorders or hormonal imbalances.

A promising method for restoring spermatogenic cell function is therapy based on the use of different types of platelet-rich plasma (PRP).

Of particular interest is the use of LP-PRP (leukocyte-poor type of platelet-rich plasma), which has a higher regenerative potential and a reduced inflammatory response due to its lower leukocyte content [3]. It is imperative to acknowledge that conventional techniques, including intracytoplasmic sperm injection (ICSI), have demonstrated limited effectiveness in men afflicted with NOA [5,6,7].

The use of growth factors in clinical practice for the treatment of skin and skeletal muscle diseases, as well as for wound healing, has recently become relevant due to their regenerative functions. It has been established that transforming growth factor (TGF-β) and epidermal growth factor (EGF) induce the proliferation and differentiation of fibroblasts, epithelial cells, immune cells, and other cell types. These factors also promote the biosynthesis of proteins, including type I collagen and fibronectin. Platelet-derived growth factor (PDGF) has been shown to regulate autocrine/paracrine function, while vascular endothelial growth factor (VEGF) has been demonstrated to stimulate angiogenesis [8]. Insulin-like growth factor-1 (IGF-1) has been identified as a potent mitogen for the majority of somatic cells [9,10,11]. The positive role of universal growth factors in fundamental biological processes in most cells (primarily proliferation and differentiation) suggests the possibility of a positive effect on spermatogenesis and its microenvironment. In preliminary studies, including those that involved human subjects, an enhancement in the quantitative and qualitative characteristics of spermatogenesis was observed in patients (as indicated by semen analysis) who received PRP treatment. Concurrently, a restoration of hormone levels, particularly FSH and LH, was observed, signifying a favorable response to the therapeutic intervention.

Researchers have demonstrated that the utilization of platelet-enriched plasma can stimulate the proliferation and differentiation of stem cells in human cell lines [12,13,14]. PRP is beginning to be used in regenerative medicine and has a positive effect, for example, in cases of impaired oocyte folliculogenesis in a model of infertile rats [15,16]. A series of experiments on female rats demonstrated the high regenerative activity of PRP in combination with low-energy extracorporeal shock wave therapy, which is known for its antifibrotic and anti-inflammatory effects in various tissues [17,18].

However, the treatment of male infertility caused by NOA remains a serious problem and requires new, promising therapeutic approaches. Given the wide range of biological effects of growth factors contained in PRP, its use in NOA can be expected to lead to the restoration of spermatogenesis and reproductive function.

It is possible that PRP components (TGF-β, IGF-1, PDGF, EGF, VEGF, and other growth factors) may activate proliferation and reduce apoptosis of spermatogenic cells, primarily stem cells and spermatogonia, as well as other important components of the testicles (Sertoli cells, Leydig cells, elements of the blood–testis barrier). The study of the role of PRP as a regenerative substrate in the restoration of spermatogenesis in men with NOA, from the perspective of assessing the life cycle of spermatogenic cells, is relevant.

The aim of this study is to evaluate intragonadal factors involved in spermatogenesis with regard to PRP administration among patients with non-obstructive azoospermia.

## 2. Materials and Methods of Research

### 2.1. Patients

All patients with non-obstructive azoospermia were young people (*n* = 41; age 22–35 years); they were physically healthy. Infectious diseases (including Mumps) and congenital anomalies of testicular development were not detected. Young people were with normal libido and sexually active. There were no allergies or hereditary history. On examination, we noted the following characteristics: male-pattern pubic hair on the body; male voice; height of 176–181 cm; and weight of 71–96 kg. Local examination (volume evaluated by Prader’s orhidometer) noted the following: both testicles in the scrotum are of normal size (the average of the right testicle is 19.1 ± 0.6 mm^3^, and the average of the left testicle is 20.1 ± 0.9 mm^3^), soft consistency, and painless.

### 2.2. Eligibility Criteria

The inclusion criteria for this study were as follows: subjects were male patients aged 21–34 years who had been diagnosed with non-obstructive azoospermia and a concomitant diagnosis of varicocele—two-sided (*n* = 21) and single-sided (*n* = 20).

The following patients were excluded from the study: those with obstructive azoospermia, infectious diseases, autoimmune diseases, or other testicular diseases.

The following criteria were used to determine exclusion from the study: the patient did not provide informed consent. We confirm that no interventions or biopsies were performed as part of the study itself.

### 2.3. Conditions

Morphological and immunohistochemical studies were conducted in the histology and immunohistochemistry laboratory of the Institute of Translational Medicine and Biotechnology at Sechenov University.

### 2.4. Duration of the Study

The study spanned a duration of six weeks. To facilitate a comparison of the morphological and immunohistochemical patterns, biopsy material from testicles with non-obstructive azoospermia was obtained on two occasions: prior to the initiation of PRP therapy and following its completion (after six weeks). The evaluation of interim checkpoints occurred on a weekly basis, with the assessment encompassing the results of blood hormone analysis (including testosterone, FSH, and LH) and the clinical observation of patients’ conditions.

### 2.5. Description of Medical Intervention (According to Medical Records/Protocols)

**Figure 1:**
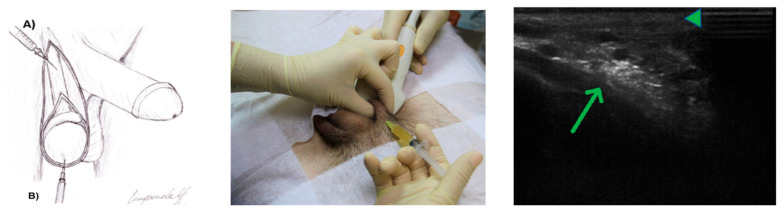
(**Left**)—Technique of PRP injections for male infertility: A—intracanalicular; B—intratesticular. (**Center**)—PRP injection technique for male infertility (intracanalicular under ultrasound control). (**Right**)—Movement of the PRP to the epididymis through 8 min (green triangle—epididymis; green arrow—intracanalicular deposition of PRP).

LP-PRP was injected under ultrasound guidance into the spermatic cord and the anterior surface of the testicle under aseptic conditions. The total injection volume was 4 mL into the spermatic cord on each side and 1 to 2 mL into the anterior surface of the testicle, depending on the volume of the testicle. Patients with a testicle volume of more than 10 cm^3^ were injected with 2 mL, while patients with a smaller volume were injected with 1 mL. The procedure was performed once a week for a period of six weeks under local anesthesia in order to minimize discomfort (Figure 1).

The LP-PRP preparation technology is predicated on a method that has been developed and described in the medical literature on the subject of the study, with adaptations made to the specifics of this work [19,20].

Blood (2 mL) was then mixed with an anticoagulant (5% sodium citrate solution) at a ratio of 1 mL of Na_3_C_6_H_5_O_7_ per 10 mL of blood. LP-PRP was obtained by two-stage centrifugation of citrated blood at room temperature (20–24 °C). In the first stage, the blood was subjected to a speed of 1800 rpm (≈543.35× *g*) for 10 min. The resulting layer of liquid in a volume of 1 mL was collected with a syringe and transferred to a clean, dry test tube. The second stage of the procedure was performed at a speed of 3400 rpm (≈1938.61× *g*) for 10 min. The superior layer was then extracted (approximately 0.85 mL), and the platelet layer that remained at the bottom (0.2 mL) was activated with calcium chloride (approximately 0.05 mL of 10% CaCl_2_). The XT-1600i system was utilized to analyze the number of blood platelets. The number of LP-PRP platelets (1,900,000 platelets/μL) was approximately 3.0 times higher than the number of blood platelets (610,000 platelets/μL). The resulting platelet-enriched plasma with a low leukocyte content (Leukocyte-Poor Platelet-Rich Plasma, LP-PRP) was administered immediately after preparation.

### 2.6. Hormone Analysis (According to Medical Records)

The concentrations of FSH and LH were measured on automatic analyzer Access-2 (Beckman Coulter, Brea, CA, USA) using Access hFSH and Access hLH reagents, respectively (Beckman Coulter, USA). The concentrations of Inhibin B (inhibin-B) and free testosterone (F-Testo) were measured using the enzyme-linked immunosorbent assay (ELISA, Helsinki, Finland) and the following reagents: CAN-FTE-260, F-Testo ELISA kit (competitive ELISA) (Diagnostics Biochem, London, ON, Canada), and LS-F4944, human inhibin βB ELISA kit (sandwich ELISA) (LifeSpan BioSciences, Seattle, WA, USA). According to the results of hormonal analysis, a slight decrease in FSH and Inhibin was found in all patients. The level of Inhibin B in the blood in group 1 before treatment was 118 pg/mL (IQR, 20.375–279.750), after treatment—144 pg/mL (IQR, 9.875–255.093). The concentration of total blood testosterone decreased from 13.91 nmol/L (IQR, 10.198–18.232) to 12.6 nmol/L (IQR, 6.812–13.255). Changes in testosterone were statistically insignificant before treatment (*p* = 0.946847) and after treatment (*p* = 0.095581).

### 2.7. Spermogram (According to Medical Records)

Semen analysis was carried out in accordance with WHO criteria (2010), ROS production in ejaculate, percentage of spermatozoa with DNA fragmentation, and MAR test (IgA%; IgG%). Based on the results of the spermogram, non-obstructive azoospermia was diagnosed in all patients.

Pathological changes in the spermogram (one or more): azoospermia; oligozoospermia (<15 million/mL); asthenozoospermia (A + B < 32%); teratozoospermia (normal forms <4%); oligospermia (<1.5 mL); cryptospermia (detection of single spermatozoa after centrifugation of ejaculate). The selection of the patient for the study was compared based on the spermogram data and FSH level.

According to the results of the spermogram, azoospermia was detected in all patients (*n* = 14) (Table 1).

### 2.8. Karyotype Analysis (According to Medical Records)

According to cytogenetic and molecular-genetic analysis (karyotype analysis, a blood test for the presence of azoospermia factor (AZF)-microdeletions, locus Y-chromosome), all the studied patients belong to the following karyotype: 46, XY- and Y-microdeletions were found in six patients (14.5%).

### 2.9. Primary Outcome of the Study

The primary objective of the present study was to assess changes in spermatogenic cell proliferation and apoptosis following PRP administration.

### 2.10. Morphological Block (Subgroup Analysis)

Samples were taken in the form of serial sections that were approximately 2–3 µm in thickness and subsequently stained with hematoxylin and eosin before being prepared for immunohistochemical examination on specialized adhesive slides.

The evaluation of the spermatogenic epithelium was carried out according to the modified Johnson scale by the following [19]: 10 points—physiological spermatogenesis; 9—disorganization of the spermatogenic epithelium, many late spermatids; 8—spermatozoa in the seminiferous tubule ≤5, isolated late spermatids; 7—spermatozoa and late spermatids absent, many early spermatids; 6—no spermatozoa or late spermatids, isolated early spermatids; 5—no spermatozoa or spermatids, many spermatocytes; 4—no spermatozoa or spermatids, isolated spermatocytes; 3—only spermatogonia; 2—absence of spermatogenic cells (sub- and/or total germ cell aplasia), only Sertoli cells; 1 point—absence of spermatogenic epithelium elements (tubular atrophy).

An immunohistochemical study was performed following the standard protocol in manual mode and included antigen demasking in citrate buffer at pH 6.0. Monoclonal antibodies to Ki-67 (ThermoFisher, Waltham, MA, USA, Clone MM1; 1:200), Bcl-2 (ThermoFisher, Clone bcl-2/100/D5; 1:50), p53 (ThermoFisher, Clone DO-7; 1:200) and Caspase 3 (ThermoFisher, Clone 74T2; 1:50), IGF-1 (Abcam, Cambridge, UK; Clone ab9572; 1:1000), TGF-β (Abcam; Clone ab215715; 1:1000), and VEGF-A (Abcam; Clone ab1316; 1:200) were employed as primary antibodies. As for secondary antibodies, universal antibodies (HiDef Detection™ HRP Polymer system, “Cell Marque”, Rocklin, CA, USA) were utilized. Secondary universal antibodies were detected using the HiDef Detection™ HRP Polymer system, produced by “Cell Marque” in the USA. For secondary antibody detection, mouse/rabbit anti-IGG, horseradish peroxidase (HRP), and DAB substrate were used. Mayer’s hematoxylin was applied beforehand to pre-stain cell nuclei.

Immunopositive cell count was performed in 10 randomly selected fields of view under a light microscope magnification of ×400 (percentage).

Microscopic analyses were conducted utilizing a video microscopy system comprising a Leica DM2000 microscope from Leica Microsystems, Wetzlar, Germany, a Leica ICC50 HD camera, and a Platrun LG computer. Morphometric data were acquired through Leica Application Suite (LAS) Version 4.9.0 image processing and analysis software.

### 2.11. Statistical Analysis

The normality of the quantitative data was ascertained through the implementation of the Shapiro–Wilk test. For normally distributed data, the group mean (M) and standard deviation (SD) were calculated. All data were presented as mean values ± standard deviation (SD). The data were processed using the SPSS 12.00 software package (Windows statistical software package; IBM Analytics, New York, NY, USA). To compare several independent groups to count cell numbers, we used one-way analysis of variance (ANOVA) with Tukey’s post hoc test. Given the study’s design, which incorporated small groups and paired comparisons (before and after LP-PRP administration), and considering the absence of normal distribution in morphometric parameters, the nonparametric Wilcoxon test for paired samples and the *t*-test for dependent samples were employed. The significance level was set at *p* < 0.05, a value that was predetermined prior to the initiation of the study.

## 3. Results

**Figure 2:**
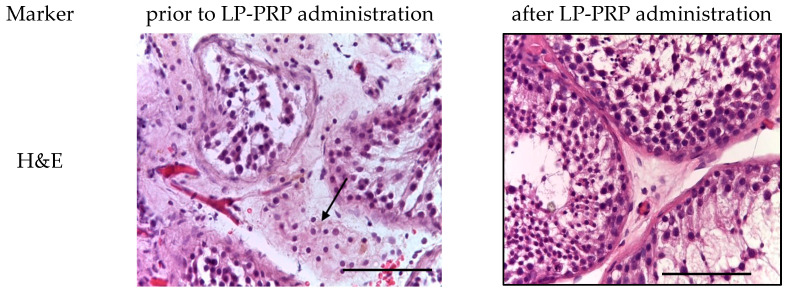
Seminiferous tubules of testicular biopsy before and after LP-PRP administration. The arrow indicates Leydig cell hyperplasia in the interstitial tissue. Staining: hematoxylin and eosin; magn.: ×400; bar: 25 μm.

A microscopic examination of testicular biopsies from patients diagnosed with non-obstructive azoospermia (*n* = 41) prior to LP-PRP administration revealed the following: A total of approximately 40 isolated seminiferous tubules were observed in each section. These tubules exhibited a rounded and elongated shape, diffusely destroyed and shrunk (i.e., underwent discomplexation), and exhibited vacuolation of the spermatogenic epithelium. The presence of signs indicative of sub- and total germ cell aplasia was also noted. The basement membrane was found to be thickened in some areas, exhibiting a moderate fibrous component (≥10 μm; weak fibrosis). The basement membrane was non-laminar and filled with secretions and protein deposits. Lamina propria is characterized by uneven thickening, which is attributed to an increase in collagen and elastic fibers. Myoid cells, exhibiting indications of dystrophy, constitute a continuous layer. In the seminiferous tubules, a pool of spermatogenic cells was identified, occupying compartments and tiers that are not characteristic of physiological spermatogenesis. The majority of cells exhibited dystrophic changes. The differentiation of typical cell associations of the spermatogenic epithelium cycle is disrupted, with type A and B spermatogonia predominating and isolated primary spermatocytes present in a single tubule. These primary spermatocytes are mitotically inactive, and spermatids and spermatozoa are undetected. Damaged seminiferous tubules with degeneration of the spermatogenic epithelium accounted for up to two-thirds of the testicular biopsy area. Concurrently, mature Sertoli cells were distinctly characterized by the structure of their nuclei and the presence of a nucleolus at the center. In the intertubular interstitial tissue, there was an observed proliferation of fibrous connective tissue and edema in certain areas. In serial sections, areas of pronounced Leydig cell proliferation (nodular hyperplasia) were identified in the interstitial tissue (Figure 2). The assessment on the S. Johnson scale indicates a range of 2–3 points.

Following the administration of PRP in all samples (*n* = 11), morphological alterations in the spermatogenic epithelium and its microenvironment were identified, exhibiting a positive trend. These alterations primarily manifested as a decline in the proportion of destructively altered seminiferous tubules and were accompanied by the emergence of all types of spermatogenic cells. A moderate proliferation of Leydig cells was observed in the interstitial tissue. In the majority of patients (*n* = 7), the Johnson score was 4 points, while in some cases, it reached 7 points (*n* = 3) and 8 points (*n* = 1). In most cases, a decrease in the severity of fibrous changes in the walls of the seminiferous tubules was visualized, which contributes to the restoration of trophic processes (Figure 2).

**Figure 3:**
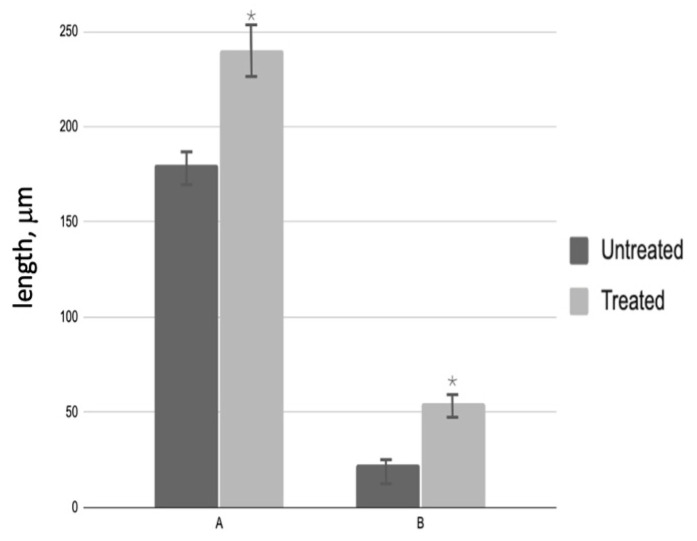
Morphological analysis of testicular biopsies: A—seminiferous tubule diameter (µm); B—germinal epithelium height (µm); * *p* < 0.05—significant difference between ‘Before’ and ‘After’ treatment groups.

The diameter of the seminiferous tubules in treated patients increased by 1.2 times compared to testicular biopsies before PRP administration. The height of the spermatogenic epithelium of testicular biopsies after PRP administration increased by 1.8 times compared to untreated patients (Figure 3).

**Figure 4:**
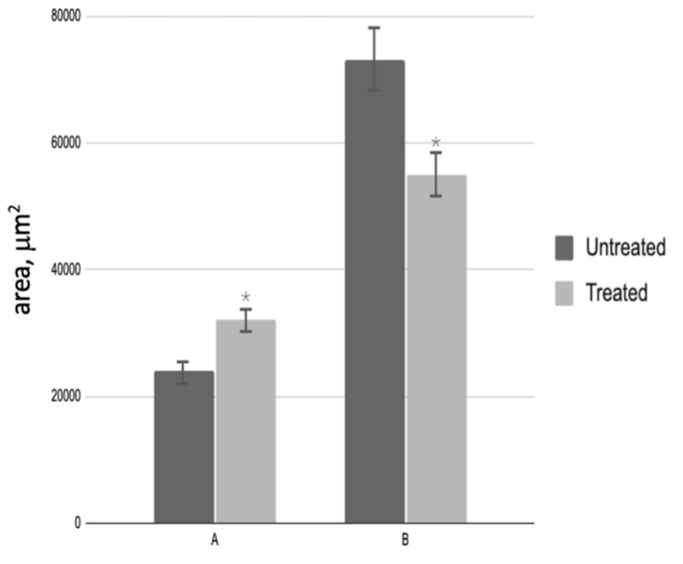
Morphological analysis of testicular biopsies: A—cross-sectional area of the seminiferous tubule (µm^2^); B—interstitial tissue area (µm^2^); * *p* < 0.05—significant difference between ‘Before’ and ‘After’ treatment groups.

**Figure 5:**
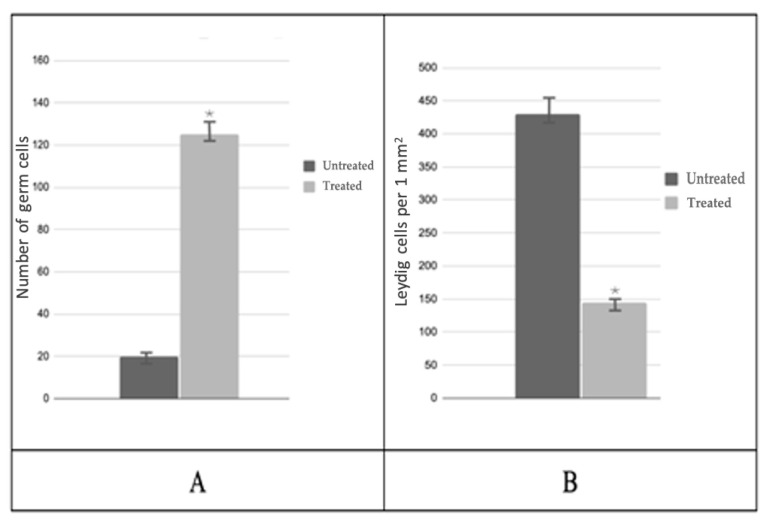
Morphological analysis of testicular biopsies: (**A**)—mean number of germ cells in the seminiferous tubule; (**B**)—number of Leydig cells per 1 mm^2^; * *p* < 0.05—significant difference between ‘Before’ and ‘After’ treatment groups.

A subsequent analysis of testicular biopsies obtained post-PRP administration revealed a notable increase in the cross-sectional area of the convoluted seminiferous tubule, which reached a magnitude of 6.0 by the conclusion of the experiment. Concurrently, a decrease in the area of interstitial tissue was observed, with a final measurement of 1.3 times that of the control group (Figure 4). The number of spermatogenic cells in most testicular samples from treated patients increased 6.7-fold (Figure 5A), while the number of Leydig cells decreased 4.1-fold compared to biopsies taken before PRP administration (Figure 5B). The quantity of Sertoli cells within these groups exhibited no alteration.

**Figure 6:**
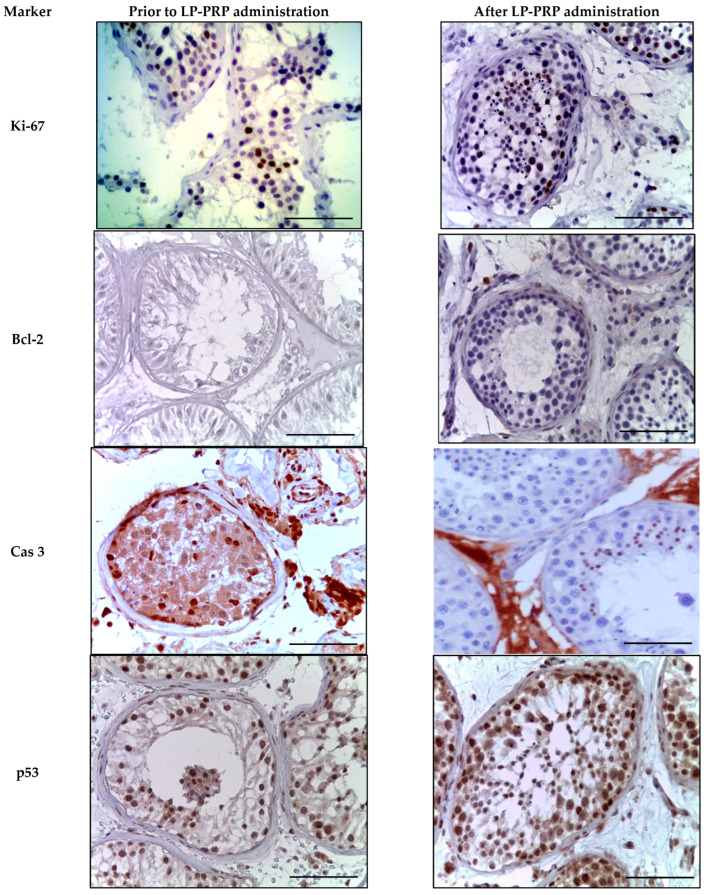
Seminiferous tubules of testicular biopsies before and after LP-PRP injection. Immunohistochemical reactions with antibodies to Ki-67, Bcl-2, caspase 3, p53 (finishing with hematoxylin); magn. ×400. Bar—25 μm.

In an immunohistochemical study against the background of LP-PRP administration, an increase in the number of Ki-67-positive cells in the spermatogenic epithelium by 2.0 times compared to the untreated patients, and a 1.5-fold decrease in the specific staining of Leydig cells were observed in the spermatogenic epithelium (Table 2, Figure 6). Bcl-2 staining was not detected in testicular biopsy specimens from either group. At the same time, single myoid cells and immunocompetent cells were Bcl-2-positive (Table 2, Figure 6). After LP-PRP administration, the number of caspase-3-positive spermatogenic cells (predominantly late spermatids) decreased 3.2-fold compared to that in untreated patients (Table 1, Figure 6). The number of p53-stained spermatogenic cells after LP-PRP administration slightly decreased compared to untreated patients, and the intensity of the reaction weakened (Table 2, Figure 6). Thus, according to the results of the immunohistochemical (IHC) examination of testicular biopsies, platelet-rich plasma (PRP) administration increased the number of spermatogenic cells stained for the proliferation marker Ki-67, while decreasing the markers of apoptosis, caspase-3, and p53.

**Figure 7:**
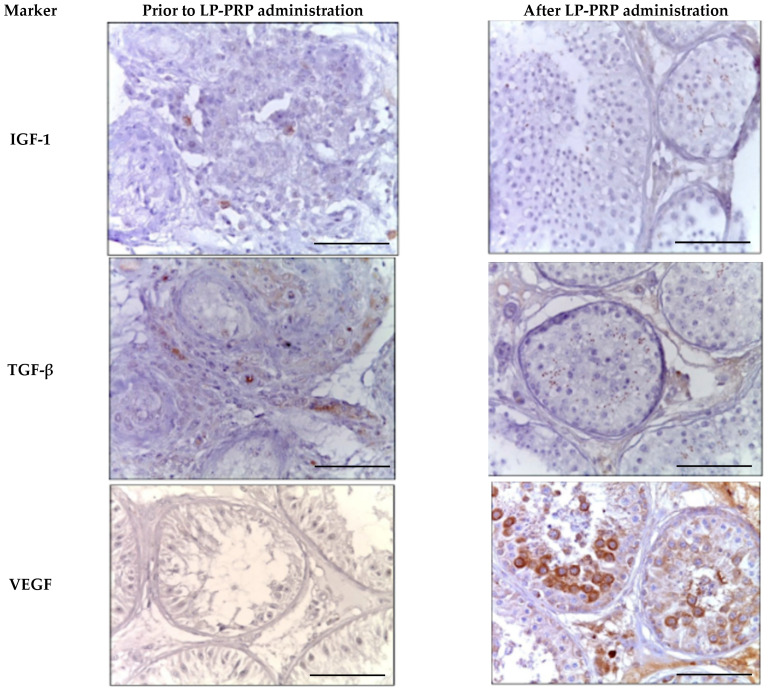
Seminal tubules of testicular biopsy specimens from patients with non-obstructive azoospermia exhibit twisted morphology before and after LP-PRP administration. The immunohistochemical reactions’ positive results for anti-IGF-1, anti-TGF-β, and anti-VEGF-A. Magn. ×400. Bar—25 μm.

During the immunohistochemical study of universal growth factors against the background of LP-PRP administration, a 2.0-fold increase in the number of IGF-1-positive cells was observed in the spermatogenic epithelium compared to untreated patients. The immunohistochemical pattern observed was mainly due to elongated spermatids and single spermatozoa. A similar immunohistochemical pattern was observed when staining with antibodies to TGF-β. Against the backdrop of repeated LP-PRP administration to patients with azoospermia, a decrease in the proportion of Leydig cells that were positive for IGF-1 and TGF-β by 2.5 times was noted (Table 2 and Figure 7)

After LP-PRP administration, the number of VEGF-A-positive spermatogenic cells increases 2.7 times in comparison with untreated patients. The cytoplasm of most primary spermatocytes and rounded spermatids undergo staining. In individuals with azoospermia, only single spermatogonia in preserved seminal tubules show IHC-positivity (Table 2, Figure 7). The administration of LP-PRP correlates with an approximately 2-fold rise in the number of Leydig cells stained for VEGF-A.

## 4. Discussion

LP-PRP was utilized as a spermatogenesis activator in patients with non-obstructive azoospermia in our study, which included universal growth factors among its constituents. These factors, as per literature data, assure the proliferative–apoptotic balance of spermatogenic cells [14].

In the clinical treatment of infertility, ICSI is the method most commonly employed, despite its drawbacks in cases of non-obstructive azoospermia, as it is invasive and ineffective [6]. Morphologists and andrologists are presented with the task of creating novel non-invasive and/or medication-based therapies. A potential solution is multiple injections of LP-PRP into the seminal tubule for patients with non-obstructive azoospermia.

Against the backdrop of LP-PRP administration, positive dynamics were observed in the main quantitative and qualitative characteristics of spermatogenesis. These dynamics included an increase in the height of the spermatogenic epithelium, which led to an increase in the area and diameter of convoluted seminiferous tubules. Additionally, there was an increase in the number of spermatogonia, spermatocytes, and spermatids, as well as the appearance of mature spermatozoa. This occurred alongside a decrease in the volume of interstitial tissue compared to biopsies from untreated patients.

Fundamental research indicates that LP-PRP enhances mitotic activity, differentiation, metabolism, and chemotaxis due to bioactive substances released from platelet α-granules. This is achieved through the stimulation of Kinase phosphorylation regulated by extracellular signal-regulated Kinases (ERK) and Akt. ERK signaling regulates cell growth, while Akt exerts anti-apoptotic effects on spermatogonia and spermatocytes [14,20].

In patients diagnosed with non-obstructive azoospermia, the histoarchitectonics of the testis were observed to significantly improve after repeated administration of LP-PRP. This improvement was manifested by noticeable increases in the area and diameter of convoluted seminal tubules, the height of spermatogenic epithelium, and the number of germ cells. Conversely, the area of interstitial tissue and the number of Leydig cells decreased significantly.

The positive effects observed with LP-PRP administration were likely due to the growth factors in LP-PRP impacting spermatogenesis.

Immunohistochemical (IHC) analysis of testicular biopsies from patients after LP-PRP administration revealed an increase in the number of cells labeled for Ki-67 and Bcl-2 proteins. This indicates increased proliferative and anti-apoptotic activity of spermatogenic epithelial cells, primarily spermatogonia and spermatocytes. Additionally, a slight decrease in apoptosis was observed and confirmed by a reduction in the number of p53- and caspase-3-stained spermatogenic cells.

The release of IGF-1 and other growth factors by platelets apparently stimulates IGFR on the surface of Leydig cells, leading to the initiation of reparative mechanisms through MAPK-, PI3K-, PLC-γ-signaling pathways. Insulin-like growth factor has demonstrated efficacy in promoting proliferation, differentiation, and migration, as well as regulating apoptosis of both somatic and germ cells. IGF and its family members facilitate conformational changes in two IGFR monomers by inducing signaling pathways, including -MAPK and -PI3K. The MAPK pathway is activated through ERK-, JNK-, and p38-proteins. Upon entering the nucleus, cytoplasmic ERK-protein regulates transcription factor activity and gene expression, leading to cell proliferation and differentiation [21,22,23]. Meanwhile, the functions of JNK and p38 aim at anti-apoptosis [21,22,24,25,26,27].

In this study, there was a significant increase in the number of spermatogenic cells stained with TGF-β after administering LP-PRP treatment compared to the untreated group. This rise is likely due to the growth factor’s dual function as a modulator. It can trigger apoptosis mechanisms in conditions with increased cellular proliferation and stimulate proliferation during apoptosis, thereby restraining excessive proliferation or apoptosis activities. We assert that the accumulation of TGF-β, which is most evident in spermatogenic cells and less significant in Leydig cells, in conjunction with the repeated administration of LP-PRP, correlates with their reaching the threshold of apoptosis. We assert that the accumulation of TGF-β, which is most evident in spermatogenic cells and less significant in Leydig cells, in conjunction with the repeated administration of LP-PRP, correlates with their reaching the threshold of apoptosis. This, in turn, triggers the activation of the anti-apoptotic mechanisms of this factor. We assert that the accumulation of TGF-β, which is most evident in spermatogenic cells and less significant in Leydig cells, in conjunction with the repeated administration of LP-PRP, correlates with their reaching the threshold of apoptosis. We hypothesize that this may be due to a higher rate of apoptosis in spermatogenic cells compared to the more stable Leydig cells prior to treatment. Thus, in Leydig cells amidst the detected compensatory hyperplasia, the requirement for TGF-β modulation of apoptosis in these cells appeared to have reduced.

Realization of beneficial outcomes is achieved through the binding of VEGF to its receptor following autophosphorylation, and the resulting activation of the PI3K/AKT- and Ras/MAPK-pathways [28,29,30,31]. Activation of these pathways leads to neovasculogenesis and an increase in the area of blood vessels within the testes. This results in improved trophicity and permeability of the blood–testis barrier, facilitating the transport of biologically active substances, while simultaneously stimulating the proliferation and differentiation of germ cells. Consequently, this promotes restoration of spermatogenesis and spermiogenesis, as well as enhances the effects of other growth factors [30,32].

The positive effects observed with LP-PRP administration are likely attributed to the impact of growth factors found in LP-PRP on spermatogenesis. IGF-1 mediates its effects through autocrine, paracrine, and endocrine pathways. It is a potent mitogen that stimulates protein synthesis and triggers the processes of proliferation and differentiation in spermatogenic epithelial cells. TGF-β maintains the balance between fibrosis and regeneration in convoluted seminal tubules. VEGF stimulates angiogenesis and enhances the trophicity of the blood–testis barrier. Meanwhile, EGF stimulates the proliferation and differentiation of the spermatogenic epithelium. PDGF supports angiogenesis, regulates matrix protein synthesis, and strengthens the proliferative activity of cells. Furthermore, FGF7, a mesenchymal cell-exclusive protein from the FGF family, is a potent mitogen for spermatogenic cells [14]. Our findings support the assumption regarding the strong efficacy of LP-PRP platelet α-granule growth factors in initiating regenerative processes. In this particular case, the treatment resulted in the restoration of the pool of spermatogenic cells.

Single studies have demonstrated that LP-PRP is the initial agent capable of preserving fertility by hindering apoptosis and re-establishing a positive antioxidant balance [33]. The positive dynamics observed in the recovery of spermatogenesis against the backdrop of repeated PRP administration can be explained by the strengthening of the antioxidant defense mechanisms of spermatogenic epithelial cells and their microenvironment. These mechanisms are known to be activated by various growth factors and other biologically active substances [33,34]. Furthermore, LP-PRP has the ability to stifle cytokine release and restrict inflammation by interacting with macrophages, enhancing tissue healing and regeneration through the promotion of new hemocapillaries [10].

Based on the literature review [34], it can be inferred that LP-PRP enhances the antioxidant defense mechanism in spermatogenic epithelial cells, thereby contributing towards the optimization of spermatogenesis.

Key mechanisms of intratesticular action of PRP in the treatment of non-obstructive azoospermia: reducing the progression of death of spermatogenic cells; stimulation of angiogenesis, increasing the vascularization index; protective effect on spermatogenic epithelium, endothelium of the blood–testis barrier; increased vascular permeability; regeneration of the stage of reproduction of spermatogenic cells: activation of mitotic division of spermatogonial cells, endothelium; cellular recruitment and migration of endothelial and myogenic cells. Restoration of the maturation stage also included the following: differentiation of spermatogenic cells due to activation of meiosis and spermatocyte metabolism; recruitment of elements of the tissue microenvironment; reduced oxidative stress activity and increased work antioxidant mechanisms; stimulation of FSH and testosterone production thanks to tropic action on Sertoli and Leydig cells, respectively.

**Figure 8:**
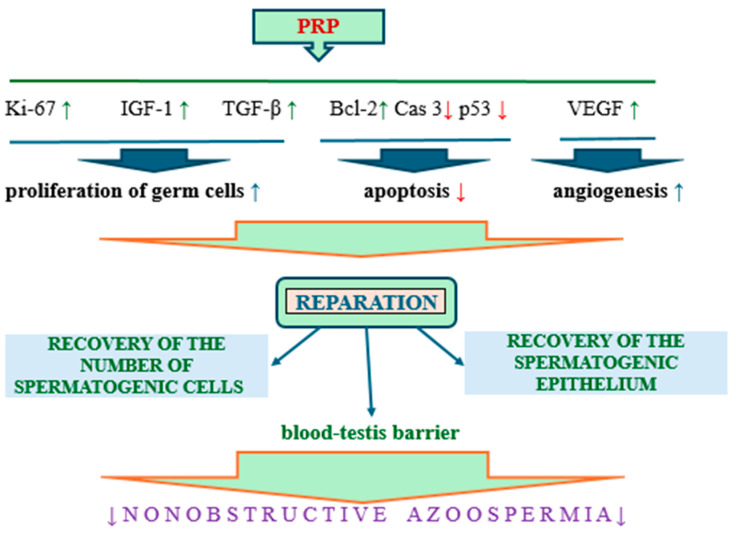
The positive effects of PRP components on spermatogenesis in patients with non-obstructive azoospermia; arrows pointing up—increase, arrows pointing down—decrease.

The positive effects of PRP components on spermatogenesis in patients with non-obstructive azoospermia are presented in Figure 8.

Thus, the components of LP-PRP, primarily growth factors, act as interstitial regulators of spermatogenesis. They mediate their effects in several ways, such as activating endocrine mechanisms against the backdrop of Leydig cell hyperplasia. This preserves the population of spermatogenic cells and shifts the proliferative-apoptotic balance towards the proliferation of spermatogenic epithelial cells.

## 5. Conclusions

The growth factors of platelet α-granules in PRP demonstrated partial or subpartial recovery of spermatogenesis and play a significant role in regulating the life cycle of spermatogenic cells. Our study in patients with non-obstructive azoospermia reveals that these factors lead to the recovery of a balanced proliferative-apoptotic state, which we confirmed through levels of Ki-67, caspase 3, and p53 immunostaining. This indicates stimulation of cell proliferation, as well as a reduction in the terminal stage of apoptosis, confirmed by a decrease in caspase-3 and p53 expression. This indicates the recovery of regenerative processes in the spermatogenic epithelium. In addition, recovery of testicular histoarchitecture was observed, including an increase in the diameter of the seminiferous tubules and the height of the spermatogenic epithelium. In human testicular biopsy samples, administration of LP-PRP resulted in a significant release of Growth factors from platelet α-granules, exhibiting their regenerative properties. This caused a marked improvement in the morphological and immunohistochemical pattern of the spermatogenic epithelium in cases of long-term nonobstructive azoospermia, which was attributed to an increase in VEGF-A, TGF-β, and IGF-1 factors.

At the same time, further research in this area, including the use of molecular genetic methods, could significantly expand our understanding of the intratesticular mechanisms regulating the life cycle of spermatogenic cells against the background of platelet-enriched plasma administration, with a view to its further application in the clinical practice of andrologists, which is important for the development of effective methods of treating patients with spermatogenesis disorders.

## Figures and Tables

**Table 1:** Spermogram.

Sign	Prior to LP-PRP Administration	After LP-PRP Administration	*p*
sperm count	51.6739 million/mL (CI95% 32.9284–70.4194)	66.5652 million/mL (CI95% 41.3588–91.7717)	*p* = 0.1373
progressively motile spermatozoa, %	32.6348% (CI95% 24.8108–40.4588)	41.3522% (CI95% 33.8687–48.8356)	*p* = 0.0474
normal morphology, %	9.3613% (CI95% 3.3096–15.4730)	11.7826% (CI95% 5.4366–18.1286)	*p* = 0.5079
viable spermatozoa, %	65.6087% (CI95% 57.8241–73.3933)	71.3478% (CI95% 66.1773–76.5183)	*p* = 0.0074
spermatozoa with fragmented DNA, %	25.3348% (CI95% 19.8673–30.8023)	15.913% (CI95% 10.6282–21.1979)	*p* = 0.0008
antioxidant action of AOT is the amount of ROS in the ejaculate	6.7191 CPM×105 (CI95% 2.4624–10.9759)	3.3427 CPM×105 (CI95% 1.4555–5.2300)	*p* = 0.0113
ejaculate volume	3.1957 mL (CI95% 2.5166–3.8747)	3.3870 mL (CI95% 2.8098–3.9641)	*p* = 0.4556

**Table 2:** Percentage of spermatogenic cells that are IHC-positive in testicular biopsies of patients with non-obstructive azoospermia before and after LP-PRP treatment at *p* < 0.05.

Marker	Before LP-PRP	After LP-PRP
Ki-67	16.0 ± 1.7	21.6 ± 1.6
Bcl-2	13.1 ± 1.2	14.2 ± 1.1
p53	81.3 ± 3.7	72.3 ± 4.6
Caspase 3	79.0 ± 4.2	63.2 ± 4.1
IGF-1	10.1 ± 3.4	25.1 ± 5.1
TGF-β	9.2 ± 3.2	29.2 ± 7.1
VEGF-A	14.1 ± 5.3	38.4 ± 3.9

## Data Availability

The study did not generate publicly available archival data.
